# Navigating new product development: Uncovering factors and overcoming challenges for success

**DOI:** 10.1016/j.heliyon.2023.e23763

**Published:** 2023-12-17

**Authors:** Mohammad Falahat, Shyue Chuan Chong, Cindy Liew

**Affiliations:** a Asia Pacific University of Technology and Innovation (APU), Malaysia; b Tunku Abdul Rahman University of Management and Technology (TAR UMT), Malaysia; c Universiti Tunku Abdul Rahman (UTAR), Malaysia

**Keywords:** New product development (NPD), Critical success factors, Challenges for new product development

## Abstract

New product development (NPD) frequently encounters failures, whether during the development phase or in the subsequent commercialization stage. These failures can often be attributed to root causes originating in the early stages of NPD, known as the front end. This study aims to investigate the factors that contribute to NPD success and the challenges faced by firms throughout the development process. To achieve this objective, an in-depth qualitative approach utilizing interviews was employed to explore the internal and external factors influencing NPD success. Additionally, the study aims to identify specific challenges encountered by firms during the NPD process. The identification of these factors and challenges are crucial for companies as it can lead to long-term cost savings.

The findings of this study have broad implications for firms regardless of their product plans. By adopting the derived approach, companies can effectively navigate the product development process and gain insights into the potential challenges associated with introducing a new product into an existing market. As customer preferences evolve with technological advancements, the barriers and challenges faced by new products entering established markets also increase. Therefore, firms that incorporate the results of this study into their product development practices across various industries can avoid pitfalls and achieve greater efficiency in terms of time and costs. This research sheds light on critical areas that contribute to successful product development and provides valuable guidance for firms striving to excel in their NPD endeavors.

## 1 Introduction

In an ever-evolving market driven by technological advancements and increasing consumer purchasing power, the pursuit of novel products and services remains constant. However, the path to success in New Product Development (NPD) projects is often fraught with challenges, leading to failure either during the later stages of development or upon commercialization. These failures are frequently rooted in the crucial initial stages of NPD, commonly known as the front end [1].

Failure of a product refers to the state or circumstance of not fulfilling the intended objective or expectations of the intended audience. Product failures occur when a new product fails to generate sufficient revenue following its launch, resulting in its eventual downfall. When a product does not pay its costs and marketing expenses, it is considered a tremendous failure. Typically, a product's failure occurs during its utilisation period [2].

Numerous factors are identified in the literature that contribute to product development failure, with an insufficient customer focus often standing as a primary concern. The best defensive strategy is to cater to consumer wants and needs. Products that do not satisfy a specific consumer demand struggle to outcompete established brands. Ineffective communication strategies supporting the launch of a new product often lead to its downfall. A product is likely to be perceived as unique if it performs a new function or an old function in a unique way, or if it holds a competitive edge in pricing and performance. Effective customer-centric companies have systems and processes in place to capture diverse customer requirements, transforming them into robust specification requirements that can guide the NPD team.

Product failure can stem from inadequate marketing planning. Effective positioning and marketing are crucial for conveying the product's benefits and competitive edge [3]. An ineffective marketing strategy can lead to the product's downfall if it fails to guide potential customers through the various stages of the buying process [4]. Despite careful planning and strategizing, a product may still fail if the marketing budget falls short or the execution of the marketing plan is flawed.

Success in the market is largely determined by optimal product launch timing. Anticipating and capitalizing on market opportunities before competitors is crucial. While being first-to-market isn't always advantageous, excessive delay or ill-timed launch may result in decreased customer demand by the time the product enters the commercialization phase [5]. It's often seen that companies launch unfinished products out of fear of falling behind, resulting in iterative versions with minor enhancements. Besides that, product failure often stems from technical product defects. Overengineering may provide a technological advantage but can also result in high costs to both the company and the consumer, thereby giving competitors an upper hand. While it's important to address technical flaws, this shouldn't come at an exorbitant cost. The repercussions of poor quality can outweigh the advantages of a new product launch, potentially inflicting devastating damage on the product's or company's brand [6,7].

This paper is set out to identify and scrutinize the pivotal factors and challenges related to new product development. Consequently, the study poses two research questions, 1. What are the factors that lead to successful product development? 2. What internal and external obstacles are encountered in the product development process?

To comprehensively answer these research questions, the study sets forth two primary objectives: the first objective is to explore the critical success factors in product development. This objective involves in-depth analysis and examination of various critical success factors influencing the outcome of product development projects. By understanding these factors, organizations can improve their NPD processes and enhance their chances of creating successful products. The second objective is to explore anticipated challenges in product development. This necessitates the identification and analysis of internal and external hurdles encountered during the different stages of product development. Understanding these challenges will help organizations anticipate and overcome potential obstacles, ensuring smoother product development processes.

This study aims to shed light on the critical factors and challenges influencing new product development success. By answering the research questions and meeting the research objectives, the study seeks to contribute valuable knowledge to the field and offer practical guidance to companies engaged in product development activities. With a comprehensive understanding of the complexities and nuances involved in NPD, organizations can effectively navigate the landscape and increase their chances of creating successful products that resonate with customers and thrive in the market.

## 2 Success factors for NPD

### 2.1 Top management commitment

Top managers or decision-makers are the ones who give a green or red light when a proposal is presented. This is the very first stage of product development that involves getting approval from the top management. If top management is closely involved in the early stages of NPD, the impetus behind new product ideas is greater. Senior management has the ability to offer resources and clarify project goals. For instance, senior managers must act as process supporters in approving, allocating, and directing the flow of the process [8]. Individual actions that cross functional boundaries can be coordinated by management.

The importance is highlighted in a paper by Ref. [9] where they investigated the Hong Kong toy sector. The critical success factors (CSFs) were examined in the four stages of the NPD process, and significant success factors were divided into four groups according to their implementation and significance. During phase I of the process of developing a new toy, it was observed that top management support and capital backing were two amongst the most frequently employed CSFs [9]. In the product development phase II, senior management commitment is still among the most widely adopted CSFs.

### 2.2 Involvement of cross-functional team

Front-end success has been characterized as requiring cross-functional collaboration [10]. One possible explanation is that cross-functional collaboration facilitates thorough analysis and reduces front-end volatility. Another possibility is that concept selection is usually conducted in meetings including representatives from various departments in the company [11]. In such gatherings, cross-functional collaboration facilitates concept assessment. Several scholars have looked at different types of cross-functional collaboration. The R&D and marketing interaction, according to Ref. [12], is the most likely instance of cooperative interdependence during the early stages of NPD. These two regions are in charge of product definition and concept, which are then distributed to the rest of the company's activities and departments. Process and manufacturing design should collaborate early in the process of NPD to ensure that the suggested items can be manufactured [13,14].

[15] found that the strength of NDP processes in the food business is largely dependent on customer involvement, exchange of information with internal and external stakeholders, and the importance of having a well-defined NPD process strategy and operational thinking.

### 2.3 Placement of structured NPD process

The importance of client interaction in the early stages of NPD is debatable. Some observers say, for example, that customers rarely offer corporations substantial or diversified information. However, the production of incorrect product is one of the main causes of NPD failure [8]. Paying close attention to the market's new requirements can give businesses ‘first-movers' advantage, which translates to high product success rates in the face of low competition. Teams that do not integrate client feedback into their product development efforts are likely to fail. Before starting product creation, companies should investigate the expectations and requirements of the client.

Before you take any large steps in business, make sure there is a market for what you are selling. There are no sales if there are no customers. Market research is a method of collecting data and information about your target audience in a methodical manner. It helps in determining the viability of your product or service before launching it on the market. It also offers you a sense of what is hot in the business and what drives consumers to convert and buy. As a result, you can plan your product or service's roadmap.

Venturing into the digital space of online customer reviews [16], harness the power of business intelligence to decipher and decode consumer sentiments. Their findings illuminate the myriad factors that shape consumer perceptions, drawing attention to the significance of online feedback in the product development landscape. In an era where digital voices resonate loudly, their methodology offers a compelling lens to view and evaluate customer preferences.

### 2.4 Project management capability

The project manager is responsible for the project's progress through its different stages, one of which is the beginning stage, which is divided into several goals to be achieved. A project manager seeks assistance, makes resource requests, and handles technological and organizational challenges. At successful firms, project managers are responsible for all of these responsibilities, according to Ref. [17]. Project managers are also responsible for defining goals, prioritizing work, and providing leadership on the front end. Product definitions are influenced by project managers.

Although no comprehensive study of the characteristics of good front-end project management has yet been conducted, existing research indicates that front-end activities can vary substantially in terms of sequencing, amount of similarity, and duration of the relative period [18,19]. This requires the front-end project manager to possess a wide range of skills.

### 2.5 Innovative ideas and service innovation

Offering a synthesized overview of new service development literature, Kitsios and Kamariotou, (2020) provide valuable insights into the trajectory and nuances of service-centric innovation. By mapping the extensive landscape, their work acts as a compass, directing firms to the key considerations, challenges, and opportunities inherent in the service development domain.

According to Ref. [20], technological advances enable the development of novel products. However, as a result of this evolution, it is difficult to generate new ideas for the organization. Nonetheless, capitalizing on recent technological advancements is critical to the successful production of a noble product [21]. Not only should ideas disrupt established paradigms, but product representations of these concepts must really bring value to customers to sell [21].

In the realm of service innovation [22], emphasize the critical role of digitization in shaping the innovation process. Their investigation spotlights both areas ripe for exploitation and avenues that beckon deeper exploration. With the digital landscape evolving at an unprecedented rate, the paper underscores that the key to effective service innovation lies in leveraging digital tools and platforms, aligning strategies with emerging technological trends, and recognizing the transformative potential of digitization.

Prior to producing a unique product description, the team gathers market needs from a range of sources [23,24]. Finally, a prototype concept idea is designed, allowing companies to assess whether extra work is required. If the choice is made to proceed, the first product concept enables the development phase's activities to be prioritised [12,17]. A drawing, a diagram, a prototype, or a mock-up can all be used to show an early concept for a product development [25].

## 3 Challenges

### 3.1 Internal challenges: complexity of teams’ project

Organizations form teams when a single individual or a group of individuals working sequentially cannot adequately complete tasks in a timely manner. To develop new products, teams must navigate an unfamiliar environment fraught with high levels of uncertainty, which serve as crucial drivers of group success [[26], [27], [28]]. On the other hand, when ambiguity and uncertainty are significant, team performance can deteriorate. According to Ref. [27], the contributing factor of ambiguity is twofold: the platform and the market. The first refers to the degree of ambiguity present during the project's design process, whilst the latter refers to the level of uncertainty created by ambiguity over the product's client desires.

According to Ref. [29] advocated regular discussion and courteous engagement, along with other things, to combat individuals' tendency to withdraw from teamwork under stressful conditions. On the other hand, setting acceptable communication styles within authorized teams can be difficult. Numerous interpersonal factors, not the least of which are those related to diversity, lead to the next issue.

### 3.2 Internal challenges: communicating across functions

While team cross-functionality can produce a range of beneficial outcomes when implemented according to the procedures mentioned in the preceding section, existing research indicates that achieving those outcomes is not simple. Functional variety, according to two distinct assessments of the literature, has a harmful effect on team performance in general, but especially during times of crisis and upheaval [30,31]. Apart from impairing team effectiveness, complexity has been linked to increased levels of discontent, attrition, sick absence, commitment, and workplace stress [32]. Their emphasis on team culture is very appropriate. Prior work by current authors and others established the crucial importance of the personal and social environment [[33], [34], [35]].

Meaningful demographic contrasts elicit judgements about an individual's perceived significance, aptitude, and potential to effectively respond to the job [36]. When these perspectives are wrong or out of control, they obstruct cross-functional learning and collaboration. Due to judgement errors, systems of influence and deference emerge within the team, limiting the supply of job knowledge [37]. Less honoured team members, who are more likely to experience decreased psychological safety [35], decreased self-efficacy, and a diminished sense of importance to the team and its task become less engaged in team tasks [35,38], and thus engage in less team-learning behavior [33,34,39].

### 3.3 Internal challenges: temporary team membership

Teams are project-based in a wide variety of fields, including research and new product development. When a project or field of inquiry presents itself, organization members are chosen based on their unique capacity to assist the endeavor. Certain members of the team will work on a project until it is completed, and then move on to the next one that requires their specialised skillset; others will work on the project for a shorter period of time. Individuals may collaborate on several projects with different people, depending on the organizational objectives. This flexible structure enables projects to be staffed by the most qualified professionals. At the same time, the transient character of the team might be troublesome; members must get to know one another before they can operate well as a team [40].

Consistent team participation, according to research, increases instructional and intrateam interaction [41]. Individuals who function in teams for an extended period of time, up to three years [42], become more efficient, most likely because teammates develop “transactive memory” [43]. Longevity is crucial for cross-functional collaboration in particular [44]. discovered that social tenure had a moderating effect on the link between diversity and conflict, with conflict being smaller in varied teams with a longer team duration.

[32] discovered that group longevity modifies the connection of diversity to organizational performance in a more recent study. As a result, firms tend to be forced to make a trade-off when utilizing short, project-based teams. On the other hand, they enable the application of the highest level of knowledge to every project. On the other hand, the frequency and duration of these proposal teams preclude the development of familiarity and understanding that comes with team longevity.

### 3.4 Internal challenges: fluid team boundaries

One reason for categorising a group of individuals as a “functional team” is to ensure that the group is “anchored,” which means that each member's role is explicitly expressed and acknowledged [45]. Individuals who work in constrained teams are more likely to have comparable time allocations than members of ad hoc teams. This enclosure generates a sense of shared identity, cohesiveness, and purpose, all of which contribute to the urge for cooperative behavior [46,[46], [47], [48]].

Two issues arise with NPD teams. To begin with, the advantage of cohesion has restrictions. Members can become so self-absorbed that they lose sight of the outer world and their own connections, compromising team effectiveness. Both environmental [[49], [50], [51]] and border [[52], [53], [54], [55]] perspectives assert that external contact with individuals outside the team makes a significant contribution to team performance [55]. established that, while correlation was used to determine that communication has no effect on team performance, interteam communication does.

Second, NPD teams rarely operate within defined exact parameters and adhere to consistent time restrictions. Typically, NPDs are made up of a core set of members who are fully responsible for group performance and depend on others to fill temporary team positions. As [33] illustrates, an NPD team may include core members from advertising, product engineering, and manufacturing, as well as part-time members with finance or legal expertise. Emphasizing on full squad participation for activities that are organically smaller than core duties is inefficient, which is why only a few NPD teams embody the “real team” notion, hence raising the coordination barrier in favour of more efficient resource utilisation.

### 3.5 Internal challenges: organizational structure

Numerous organizations fail to develop structures that support the success of teams [56]. Certain organizational structures, such as individual-based awards and department-based sharing of benefits, work against teamwork [57]. Numerous team failures are attributed to inconsistencies in task–reward structures [58]. To maximise performance and productivity, the interrelation of incentives should match the complexity of tasks [59]. Individuals or departments' contributions to the final product are barely distinguishable in the ideal form of teamwork, and as a result, all individuals should get equal credit for their team's play. This does not stop the team's “star” members from receiving further individual recognition [15]. To foster cooperation, however, organizational rewards must be pervasive, acknowledged, and appreciated such that the message communicated to team members is coherent [60].

Regardless of the fact that service innovation has a favorable influence on team performance, many firms set performance evaluation and rewards on an individual basis [59]. This may encourage team members to prioritise individual achievement and credit over collective goals, particularly when they conflict [61]. described a new product development team in which an engineer was responsible for utilizing cutting-edge technological innovation and the marketer was responsible for establishing connections with clients to identify their desires for the new product. Although these foci were consistent with the values and incentives of their functional divisions, they impeded team achievement. Therefore, the challenge to cooperation is that it operates within an environment that promotes personal achievement, but strives for collective success.

### 3.6 External challenges: price-income levels

The size of their markets justifies international firms' spending in research and development, which results in numerous new product/service developments. When these firms attempt to introduce these new products/services in poor nations, they frequently retain the majority of the product/service attributes, resulting in costs that are relatively costly for the majority of developing country consumers. In the majority of developing countries, only roughly 5–10 % of households are categorised as middle class [13]. Except for China and India, middle-class markets are typically modest. A high price may imply that the product is too advanced for the customer's needs. As a result, it may drive potential consumers to seek more relevant alternatives.

### 3.7 External challenges: technological-developmental issues

Generally, developing countries lack a strong technology foundation and trained scientists. Due to a lack of funding, many countries have very few research universities, which contributes to the shortage of highly trained scientific experts [13]. Technology is the catalyst for innovation. When a technical base is lacking, including trained researchers and funding, new product development is difficult. It is difficult for multinational corporations to justify investing in human and financial capital just for the purpose of developing products for underdeveloped countries. As a result, these corporations tend to concentrate their efforts on developing products/services for developed countries on markets capable of supporting these breakthroughs and frequently overlook developing markets.

Often, technological flaws in the product are the fundamental reason for its failure. Designers and product technologists are capable of overengineering even the most sophisticated laboratory instruments. This benefits the company's technical advantage over competitors. However, an “over-engineered” product is costly for the company and ultimately for the customer, since competitors gain an edge over the “over-engineered” product. Although technical deficiencies must be remedied, the cost of doing so should not be unacceptably high.

### 3.8 External challenges: capital constraints

Financial resources are often limited in developing countries, with the available capital primarily directed towards economic development rather than investment in products and services. International corporations primarily generate their revenues from large industrialized markets, making it challenging to justify allocating funds for research in capital-scarce developing nations. Executives of international companies in these countries often face budget constraints for research and development due to the high costs and scarcity of capital. Consequently, these leaders tend to focus their efforts on cost reduction in areas such as manufacturing, shipping, marketing, and customer service [].

Innovative organizations encounter financial barriers when it comes to investing in innovation due to externalities, informational asymmetries, and challenges related to appropriability of returns on R&D investments. These factors contribute to higher costs associated with R&D investments, leading to underinvestment in innovation activities. The cost disparity between external and internal costs can further exacerbate this underinvestment, along with limitations in liquidity. Consequently, some innovative ventures may be halted, delayed, or abandoned due to insufficient financial resources.

## 4 Materials and methods

### 4.1 Research design

In addition to conducting literature research, this study incorporates a one-to-one interview with the CEO of a Malaysia-based company who plays a pivotal role in the success of the firm's product and service development. The one-to-one interview method is widely utilized for data collection purposes. Individual interviews are recognized as a valuable approach for gathering comprehensive and in-depth data, providing valuable insights into individuals' perceptions, understandings, and experiences related to a specific phenomenon [62].

### 4.2 Data collection

Data was collected through an interview with Protenga, a leading insect-based company originating in Singapore and now operating from a pilot facility in Malaysia. Since its inception in 2018, Protenga has garnered international acclaim, carving a niche for itself as a frontrunner in Malaysia's insect industry.

The interviewee, Leo Wein, serves as both the CEO and founder of Protenga. With substantial experience in introducing new products and services to mature markets, Mr. Wein's insights are crucial to this research. As the primary informant for the company, he was the solitary participant in the interview.

Given the safety precautions necessitated by the Covid-19 pandemic, the interview was conducted virtually. A comprehensive questionnaire containing 24 items was emailed to Mr. Wein. The objective of this set of questions was to comprehend the strategies Protenga employed to surmount the hurdles encountered during its nascent stage, and how it successfully launched innovative products into the marketplace.

### 4.3 Data analysis

The analytical framework for this study will rely on thematic content analysis to dissect the data obtained from the interview. Thematic analysis is a method used in qualitative research to analyze data and identify patterns or themes within it. It is considered a foundational component of qualitative research [63].

Beyond the thematic analysis of primary interview data, the secondary data is organized in a tabulated form with checkboxes in each column. Each area is labeled according to the source of the research paper, and the record consists of a list of aspects mentioned in the publications. By utilizing checkboxes, it becomes possible to determine the variables that were most frequently mentioned as important factors in the success of new product development.

### 4.4 Background of protenga

Protenga, established in 2016 and headquartered in Singapore, operates from its pilot facility in Johor, Malaysia. The company specializes in the breeding, farming, and production of products derived from the Black Soldier Fly (BSF). In a discussion with Leo, the founder and CEO of Protenga, he delineated the company's aspiration to address the global environmental crisis. Portraying Protenga as a firm that merges insect technology with nutrition, Leo emphasized the deployment of technologically advanced insect farming systems to promote circular nutrient systems. This allows Protenga to manufacture eco-friendly protein for animal feed, as well as pet food.

The company's innovation stems from its use of the BSF lifecycle, establishing a symbiotic relationship between these insects and humans. As Leo elucidated during the interview, these insects have a dual role in the ecosystem - decomposing organic matter at the end of its life cycle and serving as a food source for other creatures. Inspired by this inherent circularity, Protenga developed technology to bring this natural cycle into the human food system. Their aim is to harness this technology to produce sustainable and high-quality feed and food products.

### 4.5 Protenga businesses

Protenga has diversified its portfolio to include three key products: fertilizer, protein, and oil. These are derived from various life cycles and processes at the company's Black Soldier Fly (BSF) farm, yielding versatile products applicable to industries such as agriculture, livestock, and aquaculture. Protenga's pricing strategy is tailored to each customer order. Products are benchmarked against market equivalents, such as fish meal (RM 4-6 k), fish oil (RM 4-6 k), and composted poultry manure (RM 400–800).

Operating as an integrated entity, Protenga covers the entire value chain, from primary resource production to supplying finished products. Their latest venture, YumGrubs, operates on a Business-to-Consumer/Direct-to-Consumer (B2C/D2C) model, directly selling their fast-moving consumer goods (FMCG) to end users. To reach their target market, digital marketing, e-commerce platforms, and content/social marketing strategies are leveraged. In terms of their Business-to-Business (B2B) offerings, Protenga adheres to traditional marketing strategies, with an emphasis on nurturing personal customer relationships which fosters trust and facilitates the formation of enduring business partnerships.

## 5 Results

**Fig. 1:**
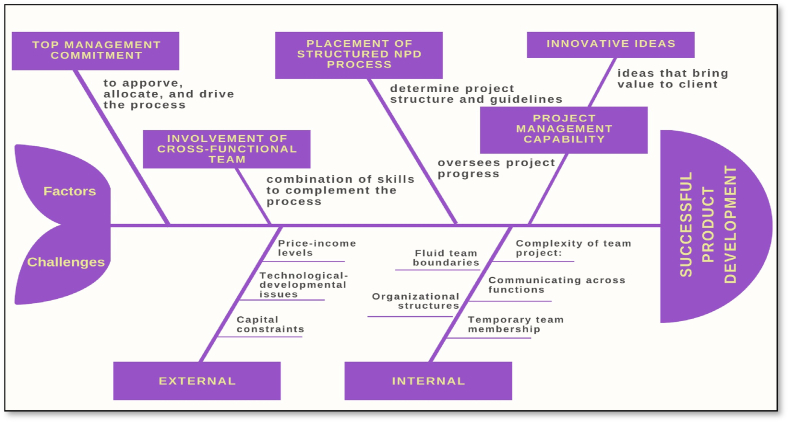
Proposed research framework of factors and challenges for product development.

Applying the thematic analysis initially resulted in the identification of 13 potential themes. However, after thoroughly examining the interview content and correlating the context with the proposed themes, the number was refined down to five key themes as below. For better illustration, please see Fig. 1.

### 5.1 Innovative idea

In response to the question, ‘What opportunity did you identify that motivated the inception of an insect-based business?’, the CEO articulated: ‘I envisioned an opportunity to design a technology that would render this natural insect-driven process amenable to the human food system, thus facilitating the production of sustainable, high-quality food and food products.’ Innovations in technology usher in new product development opportunities. The implementation of state-of-the-art technology is integral to the successful creation of superior products. The CEO concurs, asserting that the firm is offering solutions to global issues such as food shortage and excessive waste: ‘Indeed, our approach contributes to circular economy solutions within the food system.'

### 5.2 Placement of a structured NPD process

The responsibility of management in relation to the venture team is to architect the NPD framework, guidelines, and benchmarks. This strategic planning facilitates clarity for team members regarding their roles and how to navigate the NPD process. Since 2016, Protenga has executed meticulous and organized research and development in fields encompassing biology, entomology, engineering, technology, and of course, business development. The CEO affirmed: “Our production system is grounded in extensive basic and applied research conducted since 2016.” NPD approaches should underscore quality throughout the rollout phase. Moreover, these procedures must display flexibility, with the capability to merge phases, execute them simultaneously, or eliminate them upon thoughtful consideration.

### 5.3 Involvement of cross-functional teams

In response to inquiries about his management style, the CEO revealed that they have established leadership principles at Protenga.

● Utilizing Insects to Our Advantage
● Initiating Actions and Valuing Independence
● Balancing Freedom and Responsibility
● Prioritizing Purpose, Humbling Ego

From these principles, they have fostered an encouraging, inclusive, and results-oriented team culture. The CEO further elaborated, ‘We are accountable to each other, maintaining transparency and honesty in our communication.’ Cross-functional venture teams often operate with a degree of autonomy, displaying a mix of entrepreneurial attributes that enhance each other, thereby boosting process performance and outcomes. The variety of perspectives inherent in cross-functional collaboration promotes innovation.

### 5.4 Challenges: internal challenges

In response to the query regarding current challenges that require future amelioration, the CEO stated, “We have a competitive stance overall. However, being based in Malaysia, we encounter market access hurdles, for instance, to the EU, which we need to tackle in cooperation with the relevant authorities. We are still evolving and enhancing; hence our production volumes require a significant increase to reach their full potential."

For a nascent company like Protenga, constrained resources undoubtedly pose one of the greatest hurdles to scaling up production. This is not only from a financial perspective but also considering intangible assets of the company, such as employee skill sets and human resources.

### 5.5 External challenges

In discussing the challenges encountered during the export process, the CEO highlighted the complexity of differing rules and regulations imposed by various countries. What is permissible in one country may not necessarily be acceptable in another. It is therefore vital to understand the laws stipulated by the importing country to facilitate a seamless transaction.

In terms of licenses obtained by Protenga, given its production of consumable goods, the CEO confirmed, “We hold a Malaysian manufacturing license, production license for each product, sales license, and local business license. Additionally, we've acquired several free sale certificates for export."

In regards to the attitudes of Malaysians and the government towards food scarcity and excessive waste, and the actions the CEO might suggest, he said, “I'm not in a position to judge the level of seriousness attributed to these issues. However, I do see certain initiatives and policies being developed or already in place, which is promising. For instance, the government agency BioEconomy Corp is fostering the agtech and biotech field in Malaysia. Yet, we certainly hope to see more meaningful and impactful adoption of these initiatives. For instance, mandatory food waste segregation could be a significant step towards a sustainable food system, but we are still a considerable distance from that in Malaysia."

Food scarcity and excessive waste are globally critical issues, and Malaysia should give them due attention before its too late. In Europe, government approval has been granted for the use of insect proteins in pig and poultry production [64]. However, no such rules exist in Malaysia permitting the use of processed protein in any industry [65]. highlighted the challenges of producing insects for human consumption, emphasizing that stringent food safety and standard protocols must be followed. The establishment of a food-grade production facility for insect protein can be costly, and compliance certification presents a significant hurdle. Given the substantial investment and lower demand, many insect growers continue to produce animal feed and fertilizer. Therefore, if one were to produce insects fit for human consumption, food safety control is crucial, requiring the prudent handling of non-contaminated food surplus and the elimination of food waste as feed.

Table 1 represents the distribution of these critical success factors in the chosen research papers. These factors are arranged in descending order, from the most frequently discussed to the least. Top management commitment during the new product development (NPD) process emerged as the most frequently discussed factor. It is highlighted in all five selected papers, emphasizing the crucial role that top management's involvement plays in providing professional insights during the NPD process. Four factors - specific goals and milestones, cross-functional team participation, talented team members with relevant NPD experience, and a clearly defined product concept - tie for the second most frequently discussed topics, appearing in four out of the five papers. Three papers highlighted the importance of fostering an entrepreneurial culture and including user and customer participation in the NPD process. Alignment of NPD activities with overarching strategy and the availability of financial reports were each cited twice across the papers. Finally, factors such as a structured NPD process, efficient intra-organizational communication, a focus on innovation and unique ideas, and the pace of the NPD process were each mentioned once in the studied papers. Factors and challenges for product development are illustrated in Fig. 1 below.

**Table 1:** Distribution of critical success factors across relevant research papers.

	(Cooper & Kleinschmidt, 1995)	(Dwivedi et al., 2021b)	(Florén et al., 2017b)	(Cooper & Kleinschmidt, 1996)	(Lester, 1998)	Total
Top Management Commitment	✓	✓	✓	✓	✓	5
Presence of Clear Goals & Milestone Measurement		✓	✓	✓	✓	4
Involvement of Cross-Functional Teams	✓	✓	✓	✓		4
Talented Team Members with Relevant Experience to NPD Process & Activities	✓	✓		✓	✓	4
Clear Product Concept	✓	✓	✓		✓	4
Establishment of An Entrepreneurial Culture	✓	✓	✓			3
User/Customer Involvement (i.e., Market Research)		✓	✓			2
Alignment of NPD Process Activities with Strategy		✓	✓			2
Availability of Financial Requirements		✓		✓		2
Placement of Structured NPD Process		✓				1
Effective Communication Amongst Team Members & With Management		✓				1
Focusing on Innovation & Out-Of-The-Box Ideas		✓				1
NDP Process Speed		✓				1

The table above outlines a comparative study of the most frequently discussed key success factors (KSFs) for product development. Five research papers were selected at random, with the prerequisite that the titles include the keywords “Critical Success Factors” and “New Product Development”. A total of thirteen factors are listed in this comparison.

## 6 Conceptualization of findings & contributions to NPD

### 6.1 Dual-domain model of NPD challenges

One of the distinctive contributions of this paper is the identification and classification of challenges into two distinct domains: Internal and External. The Dual-Domain Model underscores the multifaceted nature of NPD, suggesting that companies must be adept at navigating challenges both from within their organizational structures and from the broader external business environment.

### 6.2 The NPD continuum of collaboration

This paper highlights the varying degrees of collaboration within NPD teams, from cross-functional collaboration to the dynamic nature of team boundaries. This continuum showcases that collaboration isn't binary; it's a spectrum. At one end, we have fixed, cross-functional teams, and on the other, fluid teams with changing membership. Recognizing where a team stands on this continuum can offer insights into potential challenges and the strategies needed to address them.

### 6.3 The sector-specific lens

NPD doesn't operate in a silo; its success and challenges are inherently tied to the sector it belongs to. By emphasizing the need to understand sector-specific variables, this paper proposes that NPD strategies must be tailored, adaptive, and sector-aware. This brings forth the idea that generalized NPD strategies might be less effective than previously thought.

### 6.4 The NPD success factors

This paper not only identifies challenges but also alludes to critical success factors. By juxtaposing challenges against success factors, we propose the NPD success model. This model can serve as a diagnostic tool for firms, helping them identify areas of strength and potential pitfalls based on internal dynamics and external market conditions.

### 6.5 The external challenge

Our findings unearth external challenges. While external factors such as technological-developmental issues can pose challenges, they can also serve as opportunities for firms to innovate and lead. Recognizing and acting upon these paradoxical opportunities can be a game-changer for firms in the NPD realm.

### 6.6 The organizational inertia dilemma

While organizational structures provide stability, they can also hinder agility, especially in the dynamic world of NPD. This paper surfaces the tension between stability and agility, suggesting that companies need to strike a balance. Too much rigidity can stifle innovation, while excessive flexibility can lead to a lack of direction.

By conceptualizing the findings in the manner above, this paper not only provides an in-depth understanding of NPD but also advances the discipline by offering novel frameworks and perspectives (see Fig. 1). These insights, grounded in rigorous qualitative analysis, have the potential to reshape how academics and practitioners approach NPD in the future.

## 7 Implications

Businesses seeking to launch new products in the market should pay close attention to certain key factors. These encompass the dedication of upper management throughout the new product development (NPD) process, participation of cross-functional teams, implementation of a structured NPD procedure, and a strong emphasis on innovation and unique ideas.

Upper management's involvement is pivotal, as they provide valuable insights and demonstrate a keen understanding of emerging market trends and opportunities. Capitalizing on these opportunities offers a competitive edge, as customers will discover your product before similar products from competitors. Establishing customer loyalty is fundamental to maintaining market competitiveness. Further, it's the duty of top management to outline essential performance indicators and milestones to monitor progress, thereby offering a comprehensive picture of areas needing improvement.

A shared objective of launching a product that caters to consumer needs should unite all departments and staff. Occasionally, conflicts may emerge as employees focus on their individual departmental goals. For example, while the finance department might aim to cut costs, the research team may need substantial investment in machinery or research and development. Even though the ultimate aim is to launch a new product, day-to-day objectives can differ across departments. It's crucial that each team or department is ready to support others when necessary.

As technology progresses, consumer demand for novel products is increasing. Major problems require innovative answers. With growing consumer buying power, they're drawn to products that engage their interest. Ideally, these products should be fresh, unique, and offer value at a reasonable price. Creating an innovative and cost-effective new product poses a significant challenge for businesses. Therefore, firms must cultivate a culture that encourages employees to tackle problems from various perspectives, thereby boosting their ability to deliver innovative solutions that add to the company's value.

## 8 Limitations and recommendations

Merely depending on the frequency of CSFs appearing in literature to demonstrate their significance in the efficiency of NPD isn't enough. It's essential to cite real-world examples from various sectors to authentically demonstrate their global success potential.

Numerous factors contribute to new product failures, including problems with the front-end activities of NPD. These can include poor handling of research results [17], erratic decision-making, and the high complexity and ambiguity that arise from competing organizational pressures. By addressing these issues, this paper aims to assist managers and their teams in pinpointing the factors that enhance the efficiency of front-end NPD operations.

While this research primarily scrutinizes internal factors that influence corporate-level new product success, it offers limited insights into how external factors can boost success rates. Considering the gaps in literature regarding NPD front-end success, it's important to further explore this domain. This requires a comprehensive understanding of the front end, including its initiation, features, conclusion, and processes. For instance Ref. [17], define the beginning of the front end as the moment when firms identify an opportunity in a semi-formal manner, suggesting that the idea—perhaps originated from an individual—needs to be communicated within the company. The incorporation of external idea sources, such as consumers and suppliers, is considered part of the front end. Although this perspective is vital, our model doesn't incorporate it; instead, it concentrates on business management.

Concerning methodology, the study includes only one respondent who also serves as the company's key informant. A more comprehensive and conclusive approach would involve multiple respondents, such as department heads, to gather more detailed and specific interview responses. Additionally, there are limited resources for examining the internal and external challenges encountered in product development. Moreover, the research discusses product development factors and challenges as a whole, without detailing strategies specific industries could implement to enhance their success probabilities. Unfortunately, no research paper can assure that adhering to the suggested steps will guarantee success in product development.

This research underscores the need to take into account both internal and external factors in corporate-level product development. Simply focusing on internal dynamics may not be enough, as external influences can markedly affect the success or failure of product development. Moreover, the case study's focus on a single sector may not fully encompass the broad spectrum of sectors in the business landscape, each with its distinct variables impacting the outcome of new product development.

The link between fundamental success determinants and project-specific success variables remains under-explored. In organizations with a culture that encourages creativity, idea refinement might unfold in innovative ways, especially when developing radically new products, even without early customer engagement or proactive environmental scanning. It remains to be clarified how the success characteristics outlined in the theoretical framework correspond with such activities and how iterations progress as a result of these actions.

## 9 Conclusions

The complexities of NPD are multifaceted and cannot be underscored enough. Through our investigation, it has become evident that a successful NPD strategy isn't solely contingent on a firm's internal strategies, processes, or cross-functional collaborations. Instead, it is intricately interwoven with a multitude of external factors that can, and often do, influence the trajectory of a product's development and its subsequent market performance.

Our research reveals that internal challenges such as communication barriers across functions, temporary team membership, fluid team boundaries, and structural issues within organizations can pose substantial impediments to the effective execution of NPD. These internal dynamics, although within a firm's control, require careful orchestration and a strategic alignment of team objectives with the broader organizational goals.

Conversely, external challenges, often outside the direct influence of organizations, add layers of complexity to the NPD process. Constraints related to price-income levels in target markets, technological and developmental disparities in certain regions, and limited financial resources in developing economies serve as poignant reminders that product development doesn't operate in a vacuum. Companies, especially those with a global reach, need to be astutely aware of these challenges, anticipating them and strategically pivoting when necessary.

Furthermore, our findings point towards the significance of acknowledging the unique variables intrinsic to different sectors within the business landscape. The challenges, risks, and potential rewards of NPD can vary dramatically from one sector to another. Hence, a one-size-fits-all approach may not yield the desired results across diverse industries.

For businesses striving to master their NPD endeavors, our research offers both a cautionary tale and a roadmap. While the path to successful product development is fraught with challenges both anticipated and unforeseen, firms equipped with a comprehensive understanding of these challenges are better poised to navigate them effectively. This study accentuates the need for organizations to adopt a holistic perspective on NPD, one that harmoniously integrates internal strategies with a keen awareness of the external environment.

## 10 Future directions of study

In future research, we advocate for a deeper exploration into sector-specific challenges and success factors in NPD. Such nuanced insights could further empower organizations to tailor their product development strategies in alignment with the unique demands and opportunities presented by their respective sectors. Besides that, one of the emergent concerns in the field of NPD, as underscored by recent literature, is the phenomenon of “Over-Featuring"—the inclination to develop products and services that exceed the genuine requirements of users, surpass market demands, and potentially strain organizational resources [66]. This trend, while rooted in the desire to offer superior value, can inadvertently lead to the creation of products that are too complex, costly, or misaligned with user needs, resulting in potential NPD failures. To counteract the challenges of Over-Featuring, future research should delve into the integration of Agile methodologies and Design Thinking. Both approaches emphasize iterative feedback and user-centricity, which can ensure products remain aligned with market needs and resonate with users.

## Declarations

All authors listed have significantly contributed to the development and the writing of this article.

## Request for publication consent

Request for permission to include company name and other information that was collected during the interview has been granted by Protenga Sdn. Bhd. For publication purposes.

## Funding statement

The authors would like to express their sincere gratitude to the Asia Pacific University of Technology and Innovation (APU) for their generous sponsorship of the Article Processing Charges (APC) associated with the publication of this paper. This support has been instrumental in enabling the dissemination of our research findings to a wider audience. We deeply appreciate Asia Pacific University's commitment to fostering academic and scientific research.

## Data availability statement

Data included in article/supp. material/referenced in article.

No additional information is available for this paper.

## CRediT authorship contribution statement

**Mohammad Falahat:** Writing – review & editing, Validation, Supervision, Conceptualization. **Shyue Chuan Chong:** Validation, Methodology, Formal analysis. **Cindy Liew:** Writing – original draft, Resources.

## Declaration of competing interest

The authors declare that they have no known competing financial interests or personal relationships that could have appeared to influence the work reported in this paper.

## References

[1] Modugu K.P. (2021). Data Anal. Mark. Entrep. Innov..

[2] Patil B.A., Kulkarni M.S., Rao P.V.M. (2019).

[3] Filip A. (2011). https://ideas.repec.org//p/pra/mprapa/31597.html.

[4] Malshe A., Hughes D.E., Good V., Friend S.B. (2022). Marketing strategy implementation impediments and remedies: a multi-level theoretical framework within the sales-marketing interface. Int. J. Res. Market..

[5] Tellis G.J., Golder P.N. (1996). First to market, first to fail? Real causes of enduring market leadership. MIT Sloan Manag. Rev..

[6] Thomke S., Fujimoto T. (2000). The effect of “front‐loading” problem‐solving on product development performance. J. Prod. Innov. Manag. Int. Publ. Prod. Dev. Manag. Assoc..

[7] Zeithaml V.A. (2000). Service quality, profitability, and the economic worth of customers: what we know and what we need to learn. J. Acad. Market. Sci..

[8] Dwivedi R., Karim F.J., Starešinić B. (2021). Critical success factors of new product development: evidence from select cases. Bus. Syst. Res. J..

[9] Sun W., Xu A., Shang Y. (2014). Transformational leadership, team climate, and team performance within the NPD team: evidence from China, Asia Pac. J. Manag..

[10] Florén H., Frishammar J., Parida V., Wincent J. (2017). Critical success factors in early new product development: a review and a conceptual model. Int. Enterpren. Manag. J..

[11] Falahat M., Lee Y.-Y., Soto-Acosta P., Ramayah T. (2021). Entrepreneurial, market, learning and networking orientations as determinants of business capability and international performance: the contingent role of government support. Int. Enterpren. Manag. J..

[12] Kohn K. (2006). Managing the balance of perspectives in the early phase of NPD: a case study from the automotive industry. Eur. J. Innovat. Manag..

[13] Chandra M., Neelankavil J.P. (2008). Product development and innovation for developing countries. J. Manag. Dev..

[14] Priest John, Sanchez Jose (2012).

[15] Sarin S., Mahajan V. (2001). The effect of reward structures on the performance of cross-functional product development teams. J. Market..

[16] Xu X., Wang X., Li Y., Haghighi M. (2017). Business intelligence in online customer textual reviews: understanding consumer perceptions and influential factors. Int. J. Inf. Manag..

[17] Khurana A., Rosenthal S.R. (1997). Integrating the fuzzy front end of new product development. Sloan Manag. Rev..

[18] Reinertsen D.G. (1999). Taking the fuzziness out of the fuzzy front end. Res. Technol. Manag..

[19] Nobelius D., Trygg L. (2002). Stop chasing the Front End process — management of the early phases in product development projects. Int. J. Proj. Manag..

[20] Cengiz E., Ayyildiz H., Kirkbir F. (2005). Critical success factors in new product development, atatürk üniversitesi sos. Bilim. Enstitüsü Derg..

[21] Lester D.H. (1998). Critical success factors for new product development. Res.-Technol. Management.

[22] Kitsios F., Kamariotou M. (2021). Service innovation process digitization: areas for exploitation and exploration. J. Hosp. Tour. Technol..

[23] Backman M., Börjesson S., Setterberg S. (2007). With concepts in the fuzzy front end: exploring the context for innovation for different types of concepts at Volvo cars. R D Manag..

[24] Kozludzhova K. (2023). Barriers to product innovations: a theoretical knowledge and research framework. Eur. J. Sustain. Dev..

[25] Dickinson J.R., Wilby C.P. (1997). Concept testing with and without product trial. J. Prod. Innovat. Manag..

[26] Eisenhardt K.M., Tabrizi B.N. (1995). Accelerating adaptive processes: product innovation in the global computer industry. Adm. Sci. Q..

[27] MacCormack A., Verganti R. (2003). Managing the sources of uncertainty: matching process and context in software development. J. Prod. Innovat. Manag..

[28] Bhuiyan N., Gerwin D., Thomson V. (2004). Simulation of the new product development process for performance improvement. Manag. Sci..

[29] Weick K.E. (1993). The collapse of sensemaking in organizations: the mann gulch disaster. Adm. Sci. Q..

[30] Bettenhausen K.L. (1991). Five years of groups research: what we have learned and what needs to Be addressed. J. Manag..

[31] Williams KatherineY., O'Reilly CharlesA. (1998). Res. Organ. Behav..

[32] Schippers M.C., Hartog N.D., Koopman P.L., Wienk J.A. (2003). Diversity and team outcomes: the moderating effects of outcome interdependence and group longevity and the mediating effect of reflexivity. J. Organ. Behav..

[33] Edmondson A.C. (1999). Psychological safety and learning behavior in work teams. Adm. Sci. Q..

[34] Edmondson A.C. (2003). Speaking up in the operating room: how team leaders promote learning in interdisciplinary action teams. J. Manag. Stud..

[35] Nembhard I.M., Edmondson A.C. (2006). Making it safe: the effects of leader inclusiveness and professional status on psychological safety and ImprovementmEfforts in health care teams. J. Organ. Behav..

[36] Berger Joseph, Fisek M. Hamit, Norman Robert Z., Wagner David G. (1998). Status Power Legitimacy.

[37] Bunderson J.S. (2003). Recognizing and utilizing expertise in work groups: a status characteristics perspective. Adm. Sci. Q..

[38] Kahn W.A. (1990). Psychological conditions of personal engagement and disengagement at work. Acad. Manag. J..

[39] Edmondson A.C., Bohmer R.M., Pisano G.P. (2001). Disrupted routines: TeamLearning and New technology ImplementationHospitals. Adm. Sci. Q..

[40] Goodman P.S., Leyden D.P. (1991). Familiarity and group productivity. J. Appl. Psychol..

[41] Moreland R.L., Argote L., Krishnan R., Tindale R.S., Heath L. (1998). Theory Res. Small Groups Soc. Psychol. Appl. Soc. Issues.

[42] Katz R. (1982). The effects of group longevity on project communication and performance. Adm. Sci. Q..

[43] Moreland R.L., Myaskovsky L. (2000). Exploring the performance benefits of group training: transactive memory or improved communication?. Organ. Behav. Hum. Decis. Process..

[44] Pelled L.H., Eisenhardt K.M., Xin K.R. (1999). Exploring the Black box: an analysis of work group diversity, conflict, and performance. Adm. Sci. Q..

[45] Hackman J.R. (1990).

[46] Ellemers N., Spears R., Doosje B. (1997). Sticking together or falling apart: in-group identification as a psychological determinant of group commitment versus individual mobility. J. Pers. Soc. Psychol..

[47] Hinds P.J., Mortensen M. (2005). Understanding conflict in geographically distributed teams: the moderating effects of shared identity, shared context, and spontaneous communication. Organ. Sci..

[48] Tyler T.R., Blader S.L. (2001). Identity and cooperative behavior in groups, group process. Intergroup Relat.

[49] Ancona D.G. (1990). Outward bound: strategies for team survival in an organization. Acad. Manag. J..

[50] Ancona D.G., Caldwell D.F. (1992). Bridging the boundary: external activity and performance in organizational teams. Adm. Sci. Q..

[51] Ancona D., Bresman H., Kaeufer K. (2002). The comparative advantage of X- teams. MIT Sloan Manag. Rev..

[52] Tushman M.L. (1977). Special boundary roles in the innovation process. Adm. Sci. Q..

[53] Gresov C. (1989). Exploring fit and misfit with multiple contingencies. Adm. Sci. Q..

[54] Tushman M.L. (1979). Work characteristics and subunit communication structure: a contingency analysis. Adm. Sci. Q..

[55] Allen T.J.J. (1984).

[56] Hackman J.R. (2002). https://www.biblio.com/book/leading-teams-setting-stage-great-performances/d/1562542056.

[57] Mumford T.V., Maynard M.T. (2020). Mines in the end zone: are there downsides to team performance?, span. J. Psychol..

[58] Ficapal-Cusí P., Enache-Zegheru M., Torrent-Sellens J. (2021). Enhancing team performance: a multilevel model. J. Clean. Prod..

[59] Wageman R., Baker G. (1997). Incentives and cooperation: the joint effects of task and reward interdependence on group performance. J. Organ. Behav..

[60] Lee F., Edmondson A.C., Thomke S., Worline M. (2004). The mixed effects of inconsistency on experimentation in organizations. Organ. Sci..

[61] Dougherty D. (1992). Interpretive barriers to successful product innovation in large firms. Organ. Sci..

[62] Ryan F., Coughlan M., Cronin P. (2009). Interviewing in qualitative research: the one-to-one interview. Int. J. Ther. Rehabil..

[63] Morgan H. (2022). Understanding thematic analysis and the debates involving its use. Qual. Rep..

[64] Pérez-Solero R. (2021). https://www.equaltimes.org/the-bug-revolution-insect-protein.

[65] Lee F. (2021). https://vulcanpost.com/747325/edible-insect-protein-farming-malaysia/.

[66] Marzi G. (2022). On the nature, origins and outcomes of over Featuring in the new product development process. J. Eng. Technol. Manag..

